# Identification and comprehensive analysis of epithelial–mesenchymal transition related target genes of miR-222-3p in breast cancer

**DOI:** 10.3389/fonc.2023.1189635

**Published:** 2023-07-20

**Authors:** Yutong Fang, Qunchen Zhang, Chunfa Chen, Zexiao Chen, Rongji Zheng, Chuanghong She, Rendong Zhang, Jundong Wu

**Affiliations:** ^1^ The Breast Center, Cancer Hospital of Shantou University Medical College, Shantou, Guangdong, China; ^2^ The Department of Central Laboratory, Cancer Hospital of Shantou University Medical College, Shantou, Guangdong, China

**Keywords:** breast cancer, miR-222-3p, epithelial-mesenchymal transition, target gene, diagnosis, prognosis, immune infiltration, drug sensitivity

## Abstract

**Background:**

Epithelial–mesenchymal transition (EMT) is a crucial mechanism that microRNA-222-3p (miR-222-3p) promotes breast cancer (BC) progression. Our study aimed to identify EMT-associated target genes (ETGs) of miR-222-3p for further analysis of their roles in BC based on bioinformatics tools.

**Methods:**

Based on bioinformatics analysis, we identified 10 core ETGs of miR-222-3p. Then, we performed a comprehensive analysis of 10 ETGs and miR-222-3p, including pathway enrichment analysis of ETGs, differential expression, clinical significance, correlation with immune cell infiltration, immune checkpoint genes (ICGs) expression, tumor mutational burden (TMB), microsatellite instability (MSI), stemness, drug sensitivity, and genetic alteration.

**Results:**

The expression of miR222-3p in basal-like BC was significantly higher than in other subtypes of BC and the normal adjacent tissue. Pathway analysis suggested that the ETGs might regulate the EMT process via the PI3K-Akt and HIF-1 signaling pathway. Six of the 10 core ETGs of miR-222-3p identified were down-expressed in BC, which were *EGFR, IL6, NRP1, NTRK2, LAMC2*, and *PIK3R1*, and *SERPINE1, MUC1, MMP11*, and *BIRC5* were up-expressed in BC, which also showed potential diagnostic values in BC. Prognosis analysis revealed that higher *NTRK2* and *PIK3R1* expressions were related to a better prognosis, and higher BIRC5 and miR-222-3p expressions were related to a worse prognosis. Most ETGs and miR-222-3p were positively correlated with various infiltration of various immune cells and ICGs expression. Lower TMB scores were correlated with higher expression of *MUC1* and *NTRK2*, and higher *BIRC5* was related to a higher TMB score. Lower expression of *MUC1*, *NTRK2*, and *PIK3R1* were associated with higher MSI scores. Higher expression of ETGs was associated with lower mRNAsi scores, except *BIRC5* and miR-222-3p conversely. Most ETGs and miR-222-3p expression were negatively correlated with the drug IC50 values. The analysis of the genetic alteration of the ETGs suggested that amplification was the main genetic alteration of eight ETGs except for *NTRK2* and *PIK3R1*.

**Conclusion:**

MiR-222-3p might be a specific biomarker of basal-like BC. We successfully identify 10 core ETGs of miR-222-3p, some might be useful diagnostic and prognostic biomarkers. The comprehensive analysis of 10 ETGs and miR-222-3p indicated that they might be involved in the development of BC, which might be novel therapeutic targets for the treatment of BC.

## 1 Introduction

Breast cancer (BC) is a prevalent malignancy among women globally, with an annual incidence of over two million cases, posing a significant threat to women’s health and life (1). The current therapeutic approach for BC patients involves a comprehensive therapeutic strategy comprising surgery, chemotherapy, radiotherapy, endocrine therapy, and targeted therapy (2). Despite the 5-year survival rate exceeding 90% for localized BC patients, the survival rate drops to <30% for those diagnosed with metastatic BC (3), which accounts for over 90% of cancer-related deaths in BC (4). Epithelial–mesenchymal transition (EMT) is a well-established process that plays a critical role in tumor-distant metastasis, whereby epithelial cells undergo a phenotypic switch to motile mesenchymal cells with heightened migratory and invasive capabilities (5). EMT has been linked to various biological properties of BC, including the acquisition of stem cell characteristics by BC cells (6). In addition, EMT has been shown to contribute to immunosuppression within the tumor microenvironment, thereby promoting tumor progression and resistance to immunotherapy (7). In addition, several lines of evidence have proven that the expression of the EMT-related gene was associated with therapeutic resistance; thus, it is necessary to evaluate the impact of EMT-related gene expression when developing a precise and individualized treatment plan for BC patients (8).

MicroRNAs (miRNAs) are a class of non-coding RNA molecules that are short and single-stranded. They function as post-transcriptional regulators of protein-coding gene expression and are involved in various cellular activities and the pathogenesis of numerous human diseases, including cancer (9, 10). Recent research has demonstrated that miRNAs can regulate the EMT process by targeting transcription factors such as Snail land Twist, thereby influencing tumor invasion and metastasis in different types of cancer (11). Certain EMT-related miRNAs have also been linked to cancer stemness and drug resistance (12).

MicroRNA-222-3p (miR-222-3p), a member of the miRNA family, is located on the human X chromosome and functions as a regulator of gene expression in the context of tumorigenesis. Specifically, miR-222-3p has been implicated in the progression of various types of cancers, acting as either a tumor suppressor or oncogene (13). In recent years, it has been established that miR-222-3p plays a significant role in promoting BC progression through the mechanism of EMT. A prior investigation has demonstrated that miR-222-3p can suppress the expression of Zinc finger E-box-binding homeobox 2 *(ZEB2)*, thereby inducing EMT (14). Moreover, recent evidence has identified *Notch3*, a member of the Notch receptor family, as a target of miR-222-3p that facilitates the EMT process in BC cells (15). Given that miR-222-3p is an EMT-associated miRNA, it is imperative to further explore the molecular mechanisms underlying its regulation of the EMT process in BC. Given the ability of a single miRNA to regulate the expression of multiple target genes concurrently (16), the objective of our investigation was to identify further potential target EMT-related genes of miR-222-3p and to explore their clinical significance, their relationship with tumor-infiltrating immune cells and drug sensitivity based on bioinformatics tools. Our findings may aid in the identification of novel drug therapy targets for patients with breast cancer.

## 2 Materials and methods

### 2.1 Data collection and analysis of differential expression and clinical significance of miR-222-3p

The level 3 normalized miRNA-sequencing data of miR-222-3p expression, including 104 normal samples and 1,103 BC tissue samples, and the clinical information of BC patients were downloaded from The Cancer Genome Atlas (TCGA) website (https://portal.gdc.cancer.gov/) (17). Then, the expression data of miRNA were then normalized to read per million (RPM) format and then were converted to log2(RPM+1). Samples lacking clinical information were excluded when analyzing the relation between expression and clinical significance in this study. The unpaired t-test was used to analyze the statistical difference between two groups of BC patients, and the Kruskal–Wallis test among more than two groups. Values of the expression level were displayed as means ± standard deviations. The differential expression of miR-222-3p was also validated by the GSE45666 dataset obtained from the Gene Expression Omnibus (GEO) database (https://www.ncbi.nlm.nih.gov/geo/) and cell lines. The receiver operating characteristic (ROC) analysis was performed by the pROC package in R to evaluate the power of miR-222-3p to differentiate BC subtypes. The Kaplan–Meier (KM) method with the log-rank test was used for analyzing the prognosis between high and low miR-222-3p expression groups with a cutoff set at the median expression level by the survival and survminer package in R. Plots were generated in R with the ggplot2 package.

### 2.2 Identification and enrichment analysis of EMT-related target genes

To identify the potential target genes of miR-222-3p, we used the miRWalk website of version 3.0 (http://mirwalk.umm.uni-heidelberg.de/) (**Supplementary Table S1**), which includes the prediction outcomes of various prediction databases (18). The EMT-related genes were obtained from the dbEMT 2.0 database (http://dbemt.bioinfo-minzhao.org/index.html) (**Supplementary Table S2**), including 1,184 genes (19). In addition, we identified the differentially expressed genes (DEGs) of the data from TCGA via the R software package limma package with the thresholds of |logFC| > 1 and false discovery rate (FDR) < 0.05 (**Supplementary Table S3**). Then, the intersection among the potential target genes of miR-222-3p, EMT-related genes, and DEGs were selected as the possible EMT-related target genes (ETGs) of miR-222-3p. To further explore the related pathways involved in the process of BC and biological functions of the possible ETGs of miRR-222-3p, we performed the Gene Ontology (GO) and Kyoto Encyclopedia of Genes and Genomes (KEGG) functional enrichment analyses using the R clusterProfiler package and visualization via the R package ggplot2. To further select the hub ETGs to improve the precision of the study, we constructed the protein–protein interaction (PPI) network of the ETGs via the STRING database (https://cn.string-db.org/) (20) and visualized via the Cytoscape (version 3.8.2), with the cytoHubba tool of which we screened 10 top hub genes as the ETGs of miR-222-3p for further research.

### 2.3 Data collection and correlation analysis of ETGs

After identifying the ETGs of miR-222-3p, we further downloaded the level 3 RNA-sequencing data in fragments per kilobase million (FPKM) format of 10 ETGs from the TCGA website. The data were converted to the format of transcripts per million (TPM) as log2(TPM+1). The Spearman’s correlation test was used to analyze the association between miR-222-3p and its ETGs and the pairwise correlation among the ETGs.

### 2.4 Differential expression and protein expression of the ETGs

The unpaired t-test was used to analyze the statistical difference of the differential expression between normal groups and BC groups of 10 ETGs with the data obtained from the TCGA and validated by the GSE45666 dataset obtained from the GEO database and cell lines. In addition, the immunohistochemistry images of BC tissues and paired adjacent normal tissues of 10 EGTs were downloaded from the Human Protein Atlas (https://www.proteinatlas.org/) to analyze the protein expression of the EGTs.

### 2.5 Cell lines and quantitative real-time PCR

MCF-7 is an ER-positive human BC cell line, and MDA-MB-231 is a human basal-like BC cell line with high invasiveness. MCF-10A is a normal breast epithelial cell line. The BC cell lines MCF-7 and MDA-MB-231, and the normal breast epithelial cell line MCF-10A were purchased from Procell (Wuhan, China) and cultured according to the manufacturer’s recommendations. Total RNA was severely isolated from the cells using the RNAsimple total RNA kit (Tiangen, Beijing, China) according to the manufacturer’s instructions. The quantitative real-time PCR (qRT-PCR) was performed using the PrimeScriptTM RT reagent kit (Takara, Japan) and the SYBR Premix Ex TaqTM II (Takara, Japan) according to the manufacturer’s instructions. Glyceraldehyde-3-phosphate dehydrogenase (GAPDH) was used as an internal reference gene, and the relative expression levels were calculated by the 2^−△△^Ct method. All specific primers are shown in **Table 1**. The Student’s t-test was used for pairwise comparison of the statistical difference between the MCF-10A cell line and BC cell lines. Plots were generated in GraphPad Prism (version 8.0).

**Table 1:** Sequences of all primers.

Primers sequence (5′–3′)	
GAPDH F	GTCAAGGCTGAGAACGGGAA
GAPDH R	TGGACTCCACGACGTACTCA
EGFR F	TCAGCTAGTTAGGAGCCCATTTTT
EGFR R	TGTGACTGAACATAACTGTAGGCT
IL6 F	ACCTAGAGTACCTCCAGAACAGAT
IL6 R	CAGGGGTGGTTATTGCATCTAGAT
SERPINE1 F	AGATTCAAGCAGCTATGGGATTCA
SERPINE1 R	TGCTGATCTCATCCTTGTTCCATG
MUC1 F	GTGAGTGATGTGCCATTTCCTTTC
MUC1 R	CCAAGGCAATGAGATAGACAATGG
NRP1 F	TTGTCTGCCCTGGAGAACTATAAC
NRP1 R	TCATGCCTCCGAATAAGTACTCTG
MMP11 F	TCGACTATGATGAGACCTGGACTA
MMP11 R	GAAAGGTGTAGAAGGCGGACATC
NTRK2 F	GAGATTGGAGCCTAACAGTGTAGA
NTRK2 R	TTCTCAGTCCCACATAAGCTTCAA
LAMC2 F	TCACCAAGACTTACACATTCAGGT
LAMC2 R	GAGATTCCGCAGTAACCTTCGATA
PIK3R1 F	TAAACCAGACCTTATCCAGCTGAG
PIK3R1 R	TCTTCATCATCTTCCACCAGTGAA
BIRC5 F	TTGCGCTTTCCTTTCTGTCAAG
BIRC5 R	CCGCAGTTTCCTCAAATTCTTTCT
MiR-222-3p F	GTTCGTGGGAGCTACATCTGGC
MiR-222-3p R	GTGTCGTGGAGTCGGCAATTC
MiR-222-3p RT Primer	GTCGTATCCAGTGCAGGGTCCGAGGTATTCGCACTGGATACGACACCCAGTA

### 2.6 Clinical significance and prognosis analysis of the EGTs

The ROC curves analysis was performed by the pROC package in R for potential diagnostic values evaluation of the up-expressed EMTs and validated by the GSE45666 dataset from GEO. To explore the clinical significance of the ETGs, we utilized the unpaired t-test to identify the association between the ETGs expression and clinical stages and PAM50 subtypes of BC. In addition, the KM method with the log-rank test was performed by the survival and survminer package in R for analyzing the prognosis of the ETGs expression, including the overall survival (OS) and the disease-specific survival (DSS), with a cutoff set at the median expression level between high and low expression groups. Plots were generated in R with the ggplot2 package.

### 2.7 Immune cell and immune checkpoint genes analysis

To explore the relationship between the ETGs expression and the immune cells in BC, we utilized the single-sample GSEA (ssGSEA) method (21) to present the infiltration enrichment of 24 common immune cells from the TCGA cohort, and the Spearman’s test was used for correlation analysis. Additionally, we applied Spearman’s correlation test to analyze the correlation between eight ICGs and the ETGs and miR-222-3p expression. Plots were generated with the ggplot2 package.

### 2.8 Tumor mutational burden, microsatellite instability, and stemness analysis

The somatic mutation data for TMB analysis were downloaded from TCGA, and the TMB scores were calculated by the observed number of mutations divided by 38Mb (22). MSI scores of the BC samples from TCGA were obtained from a previous publication (23). The one-class logistic regression (OCLR) machine-learning algorithm (24) was used for calculating the mRNA expression-based stemness index (mRNAsi) score. The unpaired t-test was used to analyze the statistical difference of the TMB, MSI, and mRNAsi scores between the high-expression and the low-expression group of 10 ETGs and miR-222-3p. Plots were generated with the ggplot2 package.

### 2.9 Drug sensitivity analysis

The R pRRophetic package was used to predict the drug response of each sample from the TCGA, and the drug sensitivity (IC50) values of each sample were estimated using Ridge’s regression with the data obtained from the Genomics of Drug Sensitivity in Cancer (GDSC) (25). Then, Spearman’s correlation test was applied to analyze the correlation between the IC50 values and the expression levels. Plots were generated in R with the ggplot2 package.

### 2.10 Genetic alteration analysis

Genetic alteration of the ETGs in the BC cohort was analyzed by cBioPortal website (http://www.cbioportal.org). Additionally, we also analyzed the OS and DSS in altered and unaltered groups.

### 2.11 Statistical analysis

The R software (version 4.2.1) and GraphPad Prism (version 8.0) were used for all statistical analyses. The above section has described detailed statistical approaches for data processing. p<0.05 was considered statistically significant.

## 3 Result

### 3.1 Differential expression and clinical significance of miR-222-3p

**Figure 1:**
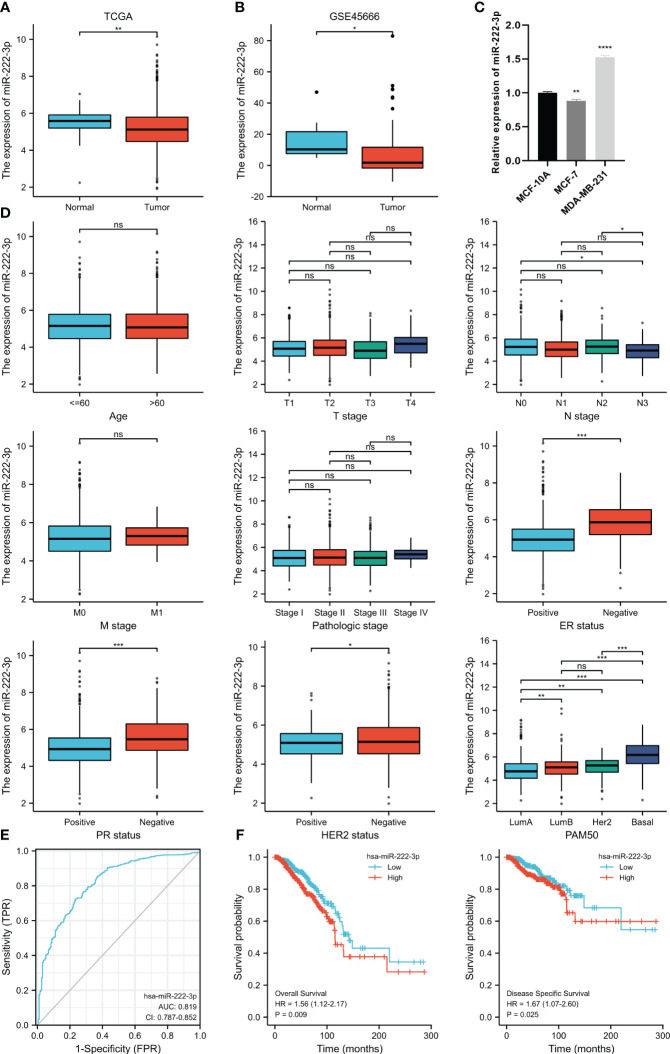
MiR-222-3p differential expression in BC and normal adjacent tissues based on TCGA database **(A)** and validated by the GSE45666 dataset **(B)** and cell lines **(C)**. The association between miR-222-3p expression and age, T stage, N stage, M stage, pathological stage, ER status, PR status, HER2 status, and PAM50 subtype **(D)**. The ROC curve shows the discriminative power between the basal-like subtype and other BC subtypes of miR-222-3p **(E)**. The KM survival curves show the OS and DSS of the high and low miR-222-3p expression groups in BC patients from TCGA **(F)**. NS, indicates no statistical difference, *p< 0.05, **p< 0.01, ***p< 0.001, ****P< 0.0001..

The analysis of the differential expression of miR-222-3p from the TCGA suggested that the expression of the BC tissues was lower than that of the normal tissues (p=0.002), which were 5.168 ± 1.117 and 5.519 ± 0.63, respectively (**Figure 1A**). The same outcome was also validated in the GSE45666 dataset (p<0.05) (**Figure 1B**). Additionally, the expression of miR-222-3p in MCF-7 was downregulated but upregulated significantly in MDA-MB-231 (**Figure 1C**). The relation between the miR-222-3p expression and the clinical indicators is shown in **Figure 1D**. The expression of miR-222-3p was associated with the status of estrogen receptor (ER), progesterone receptor (PR), human epidermal growth factor receptor 2 (HER2), lymph node status, and the PAM50 subtypes (all p<0.05). From the results, we found that BC patients with negative expression of ER and PR had a higher expression level of miR-222-3p (both p<0.001). The negative status of HER2 was associated with the high expression of miR-222-3p (p=0.031). Additionally, patients with nodal status of N0 and N2 had higher expression of miR-222-3p than patients with lymph node metastasis of N3 (p=0.021, p=0.040, respectively). Notably, the expression of miR-222-3p was 6.176 ± 1.047, significantly higher than that of luminal A (4.848 ± 1.006, p<0.001), luminal B (5.099 ± 1.025, p<0.001) and HER2-enriched subtypes (5.116 ± 0.804, p<0.001). We further evaluate the discriminative power between the basal-like subtype and other BC subtypes of miR-222-3p, and the result showed that the area under the curve (AUC) of the ROC curve was 0.819 when the cutoff value was 5.423, with a specificity of 72.5% and a sensitivity of 76.4% (**Figure 1E**). Additionally, a higher expression of miR-222-3p was correlated with better OS (HR=1.56, p=0.009) and DSS (HR=1.67, p=0.025) (**Figure 1F**).

### 3.2 Identification and enrichment analysis of EMT-related target genes

**Figure 2:**
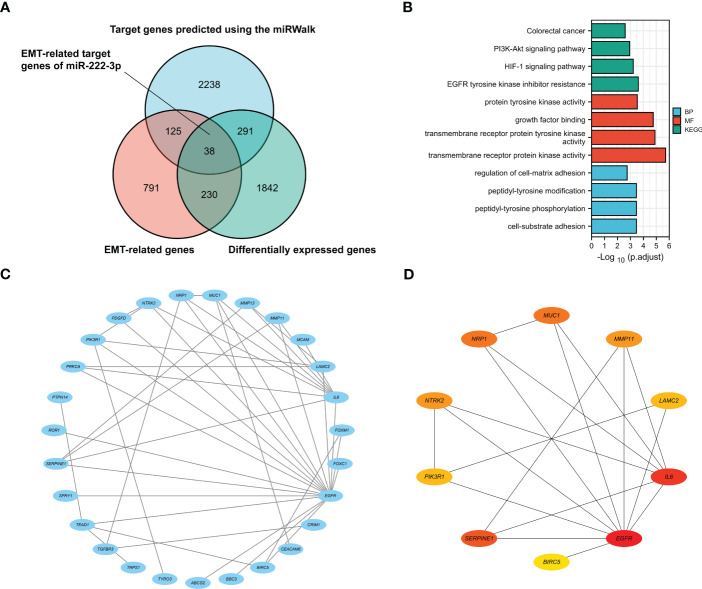
The Venn diagram shows 38 target genes as the possible ETGs of miR-222-3p **(A)**. GO and KEGG pathway enrichment analysis of 38 ETGs of miR-222-3p **(B)**. The PPI network of 38 ETGs of miR-222-3p **(C)**. Ten top hub genes of the PPI network were identified as the ETGs of miR-222-3p for further research **(D)**.

As shown in the Wayne diagram in **Figure 2A**, a total of 2692 genes were predicted as the target of miR-222-3p via the miRWalk, and 2,401 differentially expressed genes and 1,184 EMT-related genes were identified. We selected the intersection and finally identified 38 genes as the possible *ETGs* of miR-222-3p. The GO and KEGG functional enrichment analyses were performed to further explore the potential biological mechanisms of the above 38 genes (**Figure 2B**, **Supplementary Table S4**). The GO analysis showed that the ETGs might regulate the biological process of cell-matrix adhesion and intracellular signal transduction. The KEGG pathway analysis suggested that the ETGs might regulate the EMT process via the PI3K-Akt and HIF-1 signaling pathway and were associated with the drug resistance of *EGFR* tyrosine kinase inhibitors in cancer treatment. We constructed a PPI network of 38 ETGs, with 27 nodes and 42 edges (**Figure 2C**). To improve the accuracy of prediction, we identified 10 top hub genes of the PPI network as the ETGs of miR-222-3p for further research, which were *EGFR, IL6, SERPINE1, MUC1, NRP1, MMP11, NTRK2, LAMC2, PIK3R1, BIRC5* (**Figure 2D**).

### 3.3 Correlation analysis

**Figure 3:**
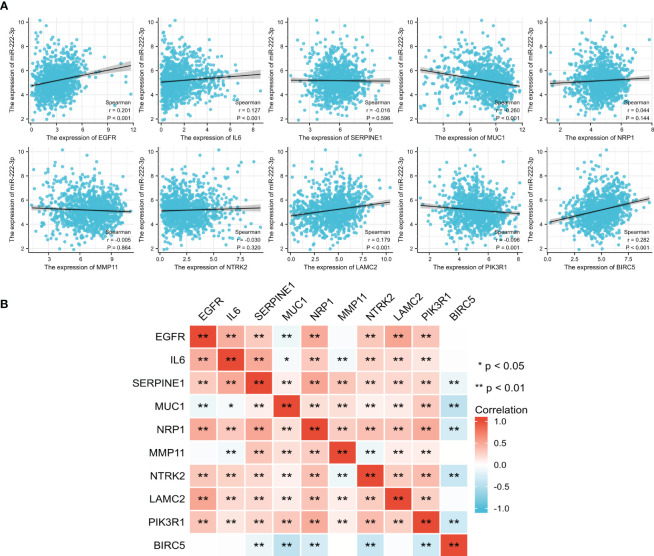
The correlation between the expression of miR-222-3p and its ETGs **(A)**. The pairwise correlation among the ETGs expression **(B)**. *p< 0.05, **p< 0.01.

We analyzed the correlation between the miR-222-3p and its ETGs, and the results of Spearman’s correlation test showed that 6 of 10 ETGs were significantly correlated with the expression of miR-222-3p. See **Figure 3A** for further details. The expression of *EGFR* (r=0.201, p<0.001), *IL6* (r=0.127, p<0.001), *LAMC2* (r=0.179, p<0.001), *BIRC5* (r=0.282, p<0.001) were positively correlated with miR-222-3p expression, and *MUC1* (r=−0.260, p<0.001) and *PIK3R1* (r=−0.096, p=0.001) negatively. Interestingly, the pairwise correlation among the ETGs showed that most ETGs had positive a correlation with others. Interestingly, the negative correlation mainly existed between *BIRC5* and other ETGs, such as *SERPINE1* (r=−0.136, p<0.001), *MUC1* (r=−0.417, p=0.003), *NRP1* (r=−0.263, p<0.001), *NTRK2* (r=−0.356, p<0.001), and *PIK3R1* (r=−0.314, p<0.001) (**Figure 3B**).

### 3.4 Differential expression of the ETGs

**Figure 4:**
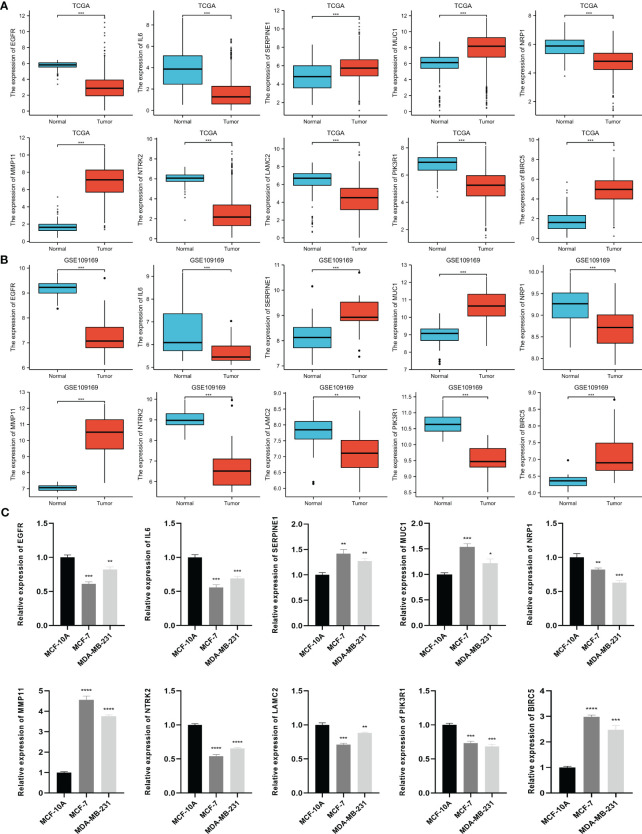
Differential expression of 10 ETGs in BC and normal adjacent tissues based on TCGA database **(A)**, which are validated by the GSE109169 dataset obtained from the GEO database **(B)** and cell lines **(C)**. *p< 0.05, **p< 0.01, ***p< 0.001, ****P< 0.0001.

**Figure 5:**
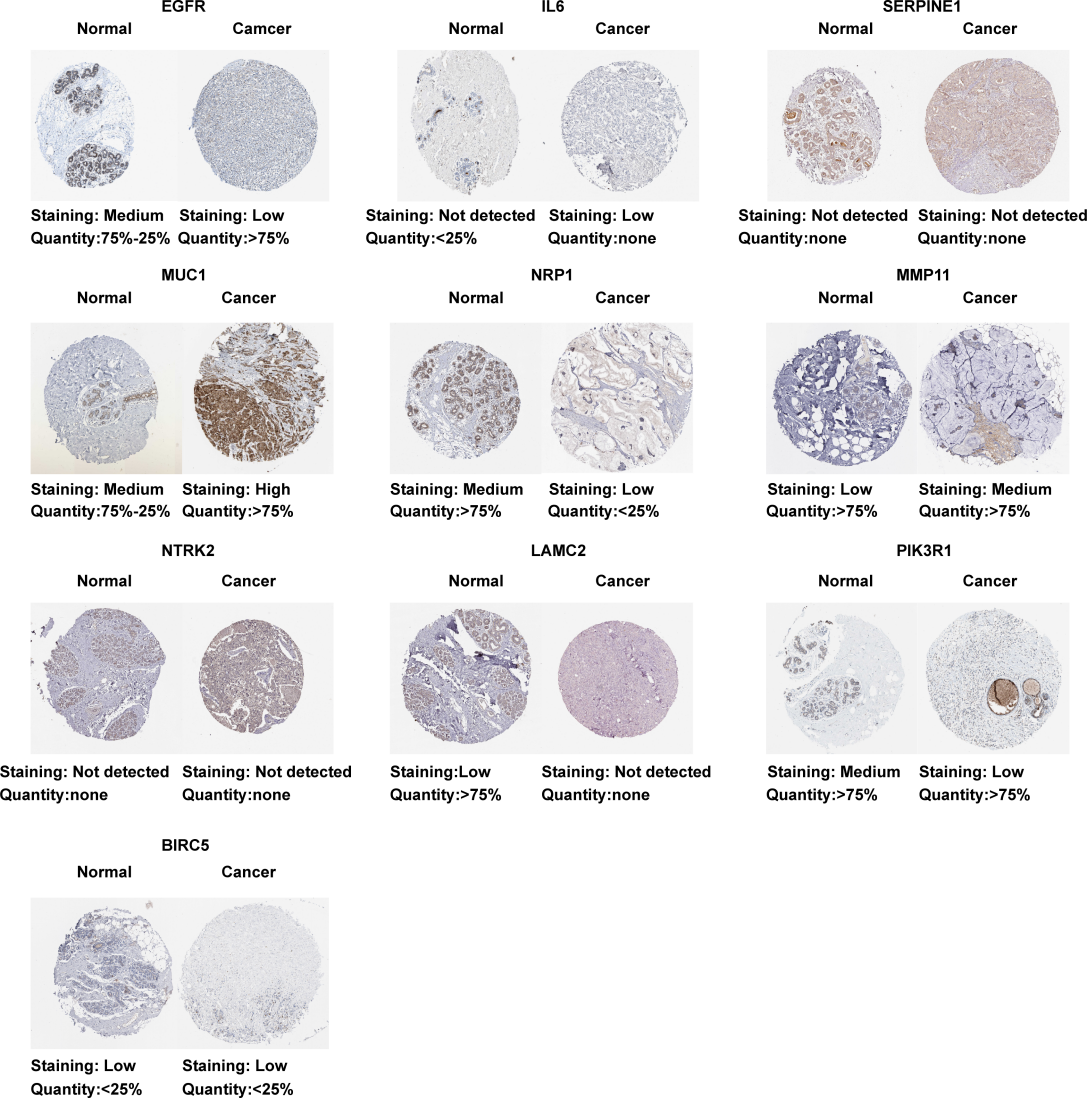
Protein expression of 10 ETGs. Images were obtained from the HPA database.

Based on the TCGA data, we analyzed the differential expression between the BC samples and the normal samples. As shown in **Figure 4A**, 7 of 10 ETGs exhibited lower expression levels in the tumor group than the normal group, which were *EGFR*, *IL6*, *NRP1*, *NTRK2*, *LAMC2*, and *PIK3R1*, and the remaining genes, *SERPINE1*, *MUC1*, *MMP11*, and *BIRC5* had higher expression level in the tumor group than the normal group (all p<0.05). The differential expression of the ETGs were also validated by the GSE45666 dataset (**Figure 4B**). In addition, the results of 10 ETGs and miR-222-3p expression were validated in two BC cells (MCF-7 and MDA-MB-231) and a breast epithelial cell line MCF-10A. The results showed that *EGFR*, *IL6*, *NRP1*, *NTRK2*, *LAMC2*, and *PIK3R1* were downregulated in BC cell lines, and *MUC1*, *MMP11*, and *BIRC5* were upregulated in BC cell lines (**Figure 4C**). The immunohistochemistry images of 10 ETGs were obtained from the HPA database to validate their protein expression. As shown in **Figure 5**, most ETGs had consistent protein expression with previous analyses in BC and normal samples of TCGA data. However, the protein expression of *SERPINE1*, *NTRK2*, and *BIRC5* showed no significant difference between BC tissue and normal tissue.

### 3.5 Diagnostic value of the up-expressed ETGs

**Figure 6:**
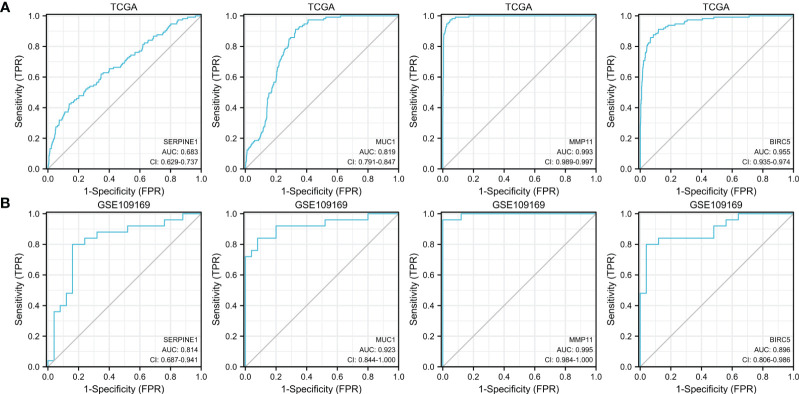
ROC curves show the diagnostic values of four up-expressed genes **(A)** and are validated by the GSE109169 dataset **(B)**.

We further evaluated the potential diagnostic values of the up-expressed ETGs for distinguishing the BC group and the normal group. As shown in **Figure 6A**, the ROC curve of *SERPINE1* had an AUC of 0.683, with a sensitivity of 42.5% and a specificity of 86.1% when the cutoff value was 4.355. The ROC curve of *MUC1* had an AUC of 0.819, with a sensitivity of 91.2% and a specificity of 67.3% when the cutoff value was 7.346. The AUC of *MMP11* was 0.993, which was the highest among the up-expressed ETGs; the sensitivity and specificity were 97.3% and 95.2%, respectively, when the cutoff was 3.461. The AUC of *BIRC5* was 0.955, with a sensitivity and specificity of 91.2% and 88.1%, respectively, when the cutoff was 3.379. The diagnostic values of the up-expressed ETGs were also validated in the GSE45666 dataset (**Figure 6B**).

### 3.6 Clinical significance of ETGs in BC

**Figure 7:**
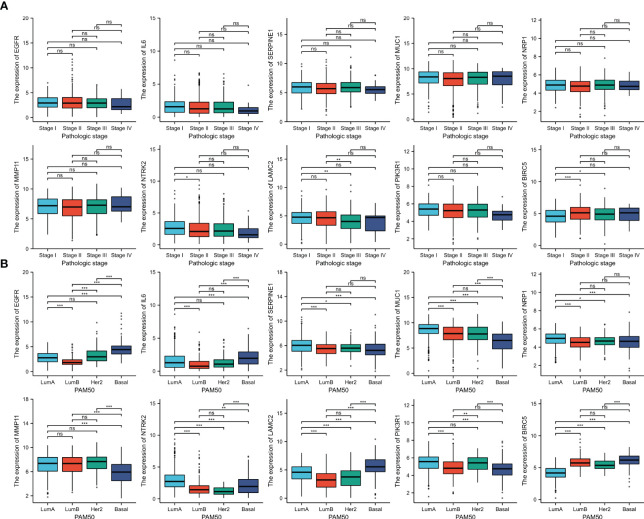
The association between 10 ETGs expression and pathologic stage **(A)** and PAM50 subtype **(B)**. NS, indicates no statistical difference, *p< 0.05, **p< 0.01, ***p< 0.001.

We analyzed the correlation of ETGs expression with clinical stages and PAM50 subtypes of BC. The results suggested that most ETGs showed no significant difference among clinical stages (**Figure 7A**). However, the analysis in **Figure 7B** suggested that the expression of most ETGs was associated with PAM50 subtypes, among which *EGFR*, *IL6*, and *LAMC2* tended to have a higher expression in the basal-like than others. Conversely, *MMP11* and *MUC1* tended to have a lower expression in the basal-like subtype. Additionally, *SERPINE1*, *MUC1*, *NRP11*, and *NTRK2* tended to have a higher expression in luminal A BC, and the expression of *BIRC5* was lower in the luminal A subtype. Interestingly, the expression of *PIK3R1* in the luminal A and HER2-enriched subtypes was higher than in the luminal B and basal-like subtypes.

### 3.7 Prognosis analysis of the EGTs

**Figure 8:**
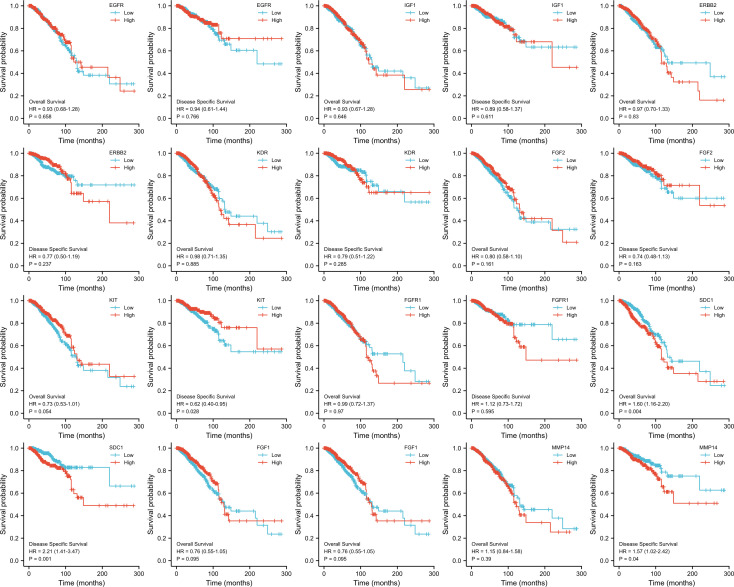
OS and DSS analysis of 10 ETGs based on TCGA.

The KM survival curves were drawn for prognosis analysis, and the results are shown in **Figure 8**. We found that BC patients with higher *NTRK2* expression had longer OS (HR=0.65, p=0.008) and DSS (HR=0.56, p=0.009). Interestingly, higher expression of *PIK3R1* was related to shorter DSS (HR=0.56, p=0.011), but OS was not significantly different. A higher expression of *BIRC5* was correlated with shorter DSS (HR=1.65, p=0.023) but not correlated with OS. The results of the KM survival curves of other ETGs were not statistically significant.

### 3.8 Immune infiltration analysis of ETGs and miR-222-3p

**Figure 9:**
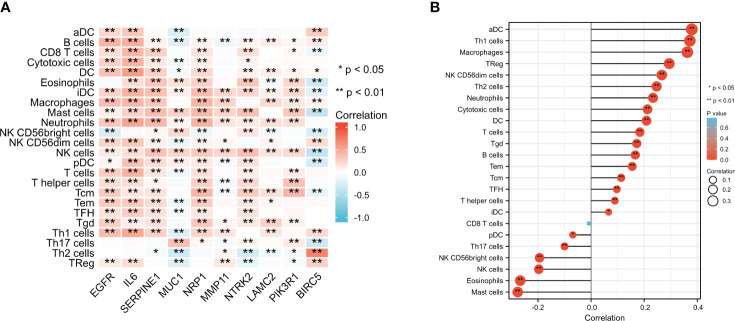
Comparison of infiltration levels in 24 common immune cells between low- and high-expression groups of 10 ETGs **(A)** and miR-222-3p **(B)**. *p< 0.05, **p< 0.01.

The immune infiltration levels of a total of 24 immune cells in BC were analyzed. The results shown in **Figure 9A** suggested that most ETGs, mainly *EGFR, IL6, SERPINE1, NRP1,* and *NTRK2*, were significantly positively correlated with various infiltration of various immune cells, among which *IL6* showed the highest positive correlation with activated DCs (aDCs) (r=0.322, p<0.001), B cells (r=0.463, p<0.001), CD8^+^ T cells (r=0.419, p<0.001), cytotoxic cells (r=0.441, p<0.001), dendritic cells (DCs) (r=0.561, p<0.001), immature DCs (iDCs) (r=0.440, p<0.001), neutrophils (r=0.515, p<0.001), NK CD56^-^ cells (r=0.328, p<0.001), plasmacytoid DCs (pDCs) (r=0.353, p<0.001), T cells (r=0.438, p<0.001), T effector memory (Tem) cells (r=0.313, p<0.001), T follicular helper (TFH) cells (r=0.299, p<0.001), and type 1 Th (Th1) cells (r=0.505, p<0.001). However, the negative correlation between ETGs expression and immune infiltration mainly existed in *MUC1* and *BIRC5*. Additionally, as shown in **Figure 9B**, the expression of miR-222-3p was positively correlated with most types of immune cells significantly, especially aDC (r=0.379, p<0.001), B cells (r=0.167, p<0.001), and macrophages (r=0.363, p<0.001). miR-222-3p expression was significantly negatively correlated with eosinophils (r=−0.267, p<0.001) and mast cells (r=−0.277, p<0.001).

### 3.9 Correlation with the ICGs expression

**Figure 10:**
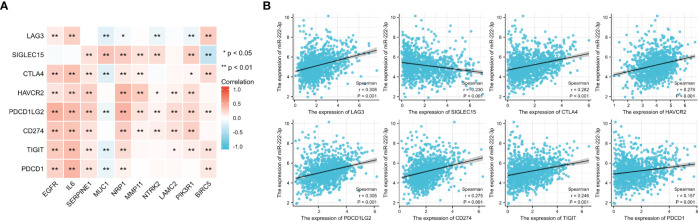
Correlation between 8 ICGs and 10 ETGs expression **(A)** and correlation between 8 ICGs and miR-222-3p expression **(B)**. *p< 0.05, **p< 0.01.

From the results shown in **Figure 10A**, we found that most ETGs of miR-222-3p were positively correlated with the expression of the *ICGs*, especially *EGFR*, *IL6*, *SERPINE1*, and *NRP1*. Interestingly, the negative correlation mainly existed between *MUC1* and ICGs, which were *LAG3* (r=−0.202, p<0.001), *CTLA4* (r=−0.213, p<0.001), *PDCD1LG2* (r=−0.125, p<0.001), *TIGIT* (r=−0.179, p<0.001), and *PDCD1* (r=−0.157, p<0.001). Additionally, miR-222-3p showed a significant correlation with eight ICGs, which were only negatively correlated with *SIGLEC15* (r=−0.230, p<0.001) (**Figure 10B**).

### 3.10 TMB and MSI analysis

**Figure 11:**
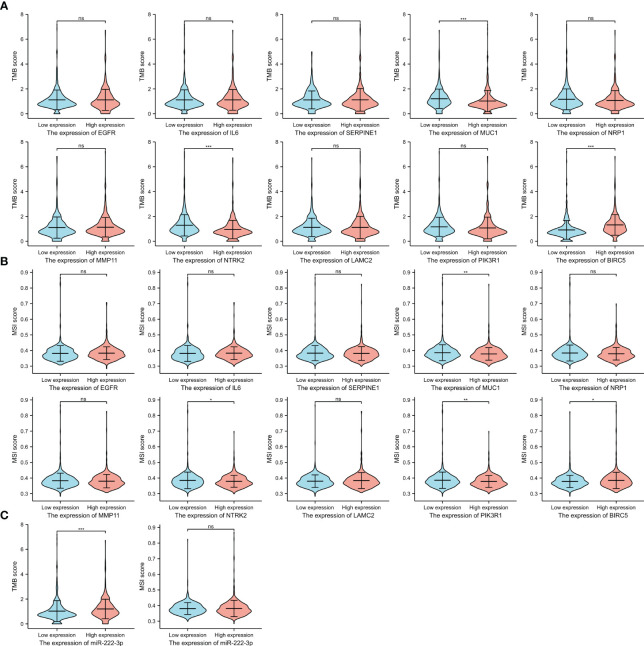
The TMB scores **(A)** and MSI scores **(B)** between the high- and low-expression groups of 10 ETGs. The TMB and MSI scores between the high- and low-expression groups of miR-222-3p **(C)**. NS, indicates no statistical difference, *p< 0.05, **p< 0.01, ***p< 0.001.

It has been suggested that patients with a high level of TMB tend to benefit from immunotherapy. **Figure 11A** showed the difference between high- and low-expression groups, and the results of the unpaired t-test showed that the lower TMB scores were correlated with higher expression of *MUC1* (p<0.001) and *NTRK2* (p<0.001), and high *BIRC5* expression was associated with a higher TMB score (p<0.001). We also evaluated the association of MSI scores between high- and low-expression groups of ETGs (**Figure 11B**), and we found that lower expression of *MUC1* (p=0.005), *NTRK2* (p=0.046), and *PIK3R1* (p=0.005) were associated with higher MSI scores, and higher *BIRC5* expression was related to higher MSI score (p=0.019). Additionally, we found that patients with higher miR-222-3p expression tend to have higher TMB scores (p=0.001) (**Figure 11C**).

### 3.11 Stemness analysis

**Figure 12:**
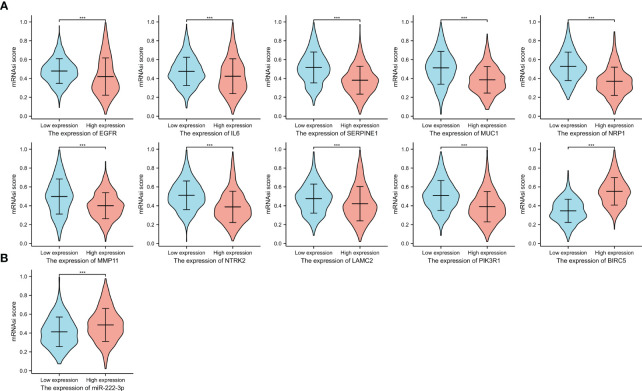
The mRNAsi scores between the high and low expression groups of 10 ETGs **(A)**, and the mRNAsi scores between the high and low expression groups of miR-222-3p **(B)**. ***, indicates P< 0.001.

We evaluated the difference in mRNAsi score between high- and low-expression groups. **Figure 12** showed that higher expression of ETGs was associated with lower mRNAsi score, except *BIRC5* conversely (all p<0.001). Additionally, BC patients with higher expression of mir-222-3p tend to have a higher mRNAsi level (p<0.001).

### 3.12 Drug sensitivity analysis

**Figure 13:**
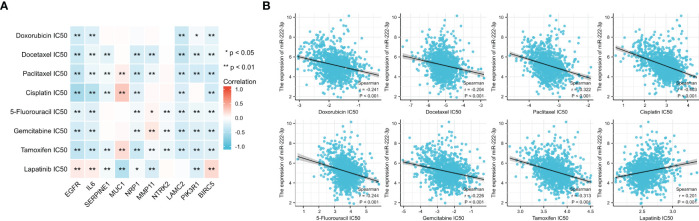
The association between the IC50 of eight drugs and ETGs expression **(A)**, and the association between the IC50 of eight drugs and miR-222-3p expression **(B)**. *p< 0.05, **p< 0.01.

The IC50 value was a value that was used to evaluate the sensitivity of drug treatment. **Figure 13A** showed the association between the IC50 of eight drugs and ETGs expression, from which we found that most ETGs were negatively correlated with the IC50 values. The positive correlation mainly existed between drug IC50 values and *MUC1*, which exhibited the highest correlation with IC50 of paclitaxel(r=0.170, p<0.001), cisplatin (r=0.313, p<0.001), and tamoxifen (r=0.203, p<0.001). In addition, in **Figure 13B**, we found that seven drug IC50 were negatively correlated with miR-222-3p expression (all p<0.001), and only the IC50 value of lapatinib was positively correlated with miR-222-3p expression (r=0.201, p<0.001).

### 3.13 Genetic alteration of ETGs

**Figure 14:**
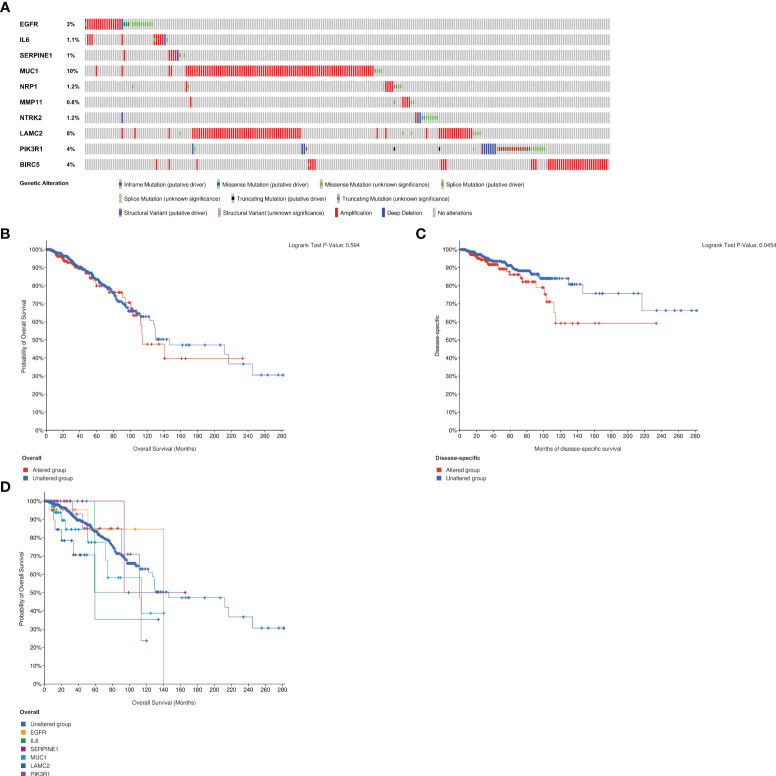
Analysis of genetic alteration of 10 ETGs **(A)**. The KM survival curves show the OS and DSS between the ETGs altered group and the unaltered group **(B, C)**. The KM survival curve shows the OS of the ETGs-unaltered group and the ETGs-altered groups of EGFR, IL6, SERPINE1, MUC1, LAMC2, and PIK3R1 **(D)**.

The analysis of genetic alteration of the ETGs shown in **Figure 14A** suggested that amplification was the main genetic alteration of nine ETGs except for *NTRK2* and *PIK3R1*. The genetic alteration rate of *MUC1* was highest among 10 ETGs, up to 10%. There was no difference between the ETGs altered group and the unaltered group in OS (**Figure 14B**), but the DSS of the unaltered group was longer than that of the altered group (p<0.05) (**Figure 14C**). The median months overall (95% CI) of *NRP1*, *MMP11*, *NTRK2*, and *BIRC5* were not applicable; thus, we analyzed the OS of the unaltered group and the ETGs-altered groups of *EGFR*, *IL6*, *SERPINE1*, *MUC1*, *LAMC2*, and *PIK3R1* (**Figure 14D**). The median months overall (95% CI) of the unaltered group was 146.50, which was longer than that of six ETG-altered groups.

## 4 Discussion

While the prognosis for patients diagnosed with early-stage BC is generally favorable, the treatment of metastatic breast cancer poses a significant challenge to public health due to its unfavorable prognosis. The process of EMT plays a critical role in tumor metastasis (5) and has garnered increasing attention in recent years. Recent research has demonstrated that multiple microRNAs are involved in the progression of BC by regulating EMT through different signaling pathways mediated by various transcription factors, thereby disabling tumor-suppressing or tumor-promoting effects, which might also be served as therapeutic molecules for the treatment of BC (11, 26). Prior research has demonstrated that miR-222-3p functions as a regulator of EMT in BC (14, 15, 27). The present investigation employs bioinformatic analysis to identify 10 fundamental ETGs of miR-222-3p for further investigation of the underlying regulatory mechanisms.

The current investigation utilized the TCGA database to conduct an analysis, which revealed that miR-222-3p exhibited a comparatively reduced expression level in contrast to normal paracancerous tissues. This finding was subsequently confirmed through qRT-PCR in MCF-7 cell lines. Nevertheless, prior research has indicated that miR-222-3p tends to exhibit a relatively elevated expression in BC tissues (28, 29). The incongruity in the outcomes can be primarily attributed to the limited sample size and regional disparities in the selection of BC patients, predominantly in Asia, in the earlier studies. Our findings indicate a significant elevation in miR-222-3p expression in MDA-MB-231 cell lines. Furthermore, the PAM50 subtype analysis revealed that miR-222-3p exhibited significantly higher expression in the basal-like subtype compared to other BC subtypes and normal tissues, which is consistent with previous studies (14, 15, 30). The AUC of the ROC curve for distinguishing basal-like BC and other subtypes was 0.819, suggesting that miR-222-3p may be serve as a specific biomarker of basal-like BC. Furthermore, the clinical implications of miR-222-3p indicate that its heightened expression is inversely correlated with negative status of ER, PR, and HER2 statuses. Specifically, research has demonstrated that miR-222-3p overexpression directly inhibits ER translation, while ER can suppress miR-222-3p expression by enlisting the nuclear receptor corepressor *(NCoR)* and thyroid hormone receptor *(SMRT)* (31). However, the mechanism of interaction between miR-222-3p and PR or HER2 remains unexplored. In addition, our survival analysis revealed that breast cancer patients exhibiting elevated expression levels of miR-222-3p were more likely to experience a poorer prognosis, thus providing further evidence that heightened miR-222-3p expression is linked to increased invasion in BC. Notably, our study also demonstrated, for the first time, that miR-222-3p expression is correlated with a broad range of immune cell infiltration and ICGs, suggesting that it may play a role in regulating the immune microenvironment during the progression of BC. TMB was proposed for efficacy predictions of immunotherapy as a marker (32), and our analysis revealed that BC patients who exhibit elevated expression levels of miR-222-3p may experience greater advantages from immunotherapy. Additionally, our findings indicate a positive correlation between miR-222-3p expression and mRNAsi scores, which suggests that BC patients with high expression of miR-222-3p are more likely to have lower degrees of differentiation and higher levels of cell stemness.

In order to gain a deeper comprehension of the fundamental mechanisms governing the EMT process, a set of 38 genes associated with EMT were identified as the potential targets of miR-222-3p. Through pathway enrichment analysis reveal, it was determined that miR-222-3p may regulate EMT via the PI3K-Akt and HIF-1 signaling pathways. Previous research has indicated that the activated PI3K-Akt signaling pathway plays a direct role in inducing EMT by upregulating the expression of Snail and phosphorylated Twist and also collaborates with other signaling pathways to facilitate EMT either directly or indirectly during the progression of cancers (33). Moreover, it has been demonstrated that the upregulation of hypoxia-inducible factor 1 *(HIF-1)* in breast cancer is linked to metastasis as an EMT activator through the mediation of EMT-related signaling pathways, transcription factors, and inflammatory cytokines (34). In this study, we constructed the PPI network of 38 EMT-related genes and subsequently identified 10 top hub genes as the ETGs of miR-222-3p for further research.

Epidermal growth factor receptor *(EGFR)* is a transmembrane protein with tyrosine kinase activity that governs cellular functions, and miR-222-3p has been reported as a downstream modulator of the *EGFR* signaling pathway that regulates EMT as a promotor (35, 36). In this investigation, the top core ETG of miR-222-3p was identified as *EGFR*, and a positive correlation of expression between the two was established. This suggests the possibility of a positive feedback loop between activated EGFR and upregulated miR-222-3p, which may promote EMT in BC. Interestingly, our findings indicate that the *EGFR* expression in BC tissues is lower than that of normal adjacent tissues. Previous studies have reported that EGFR overexpression was detected in only 15%–30% of BC but at least half of basal-like BC (36, 37). Our investigation also found that *EGFR* expression in basal-like BC was significantly higher than in other subtypes, revealing that the overexpression of which is associated with BC invasion. Furthermore, our investigation revealed a positive correlation between elevated *EGFR* expression and a majority of immune cell infiltrates and ICGs, indicating that individuals with basal-like BC who exhibit high *EGFR* expression may derive greater therapeutic benefit from immune checkpoint inhibitor therapy. Recently, the advent of *EGFR*-targeted chimeric antigen receptor T cell therapy has shown promising results in treating Basal-like BC (38). These findings underscore the potential of *EGFR* as a viable therapeutic target for basal-like BC.

Interleukin-6 *(IL6)* is a pro-inflammatory cytokine that is secreted by different cell types and is known to modulate the growth and differentiation of BC (39, 40). Studies have shown that adipocyte-secreted *IL-6* can induce EMT in BC by triggering signal transducer and activated of transcription 3 *(STAT3)* (41). Interestingly, our analysis revealed that IL6 exhibited the strongest positive correlation with many types of immune cell infiltration across multiple ETGs, suggesting its potential role in immune modulation within the tumor microenvironment. Actually, immune cells in the tumor microenvironment of most cancers were regulated by *IL6* to promote chronic inflammation to help angiogenesis for tumors (42).

Serine protease inhibitor clade E member 1 *(SERPINE1)*, also known as PAI1, is an inhibitor of the plasminogen/plasminase system, the upregulation of which was identified as a biomarker for predicting poor outcome and associated with the EMT process in BC (43, 44). Our findings indicate that the expression of *SERPINE1* was higher in BC tissues, particularly in luminal A BC. However, we did not observe a significant association between SERINE1 expression and prognosis, although the high-expression group tended to have a worse prognosis but without statistical significance. It has been reported that BC patients with high levels of *SERPINE1* may benefit from adjuvant chemotherapy (43). In our study, we found a negative correlation between *SERPINE1* expression and IC50 values of docetaxel, paclitaxel, cisplatin, tamoxifen, and lapatinib, suggesting that BC patients with high levels of *SERPINE1* might receive a better treatment effect of chemotherapy.

Mucin 1 *(MUC1)*, also known as CA15-3, is a transmembrane heterodimeric glycoprotein that is aberrantly overexpressed in BC and serves as a serum diagnostic biomarker for BC (45, 46). In the present study, we evaluated the diagnostic value of the elevated *MUC1* expression and found it to be favorable, with a sensitivity of 91.2% and a specificity of 67.3%. Previous research has demonstrated that *MUC1* can facilitate *IGF-1*-induced EMT in the MCF-7 breast cancer cell line and induce tamoxifen resistance in ER-positive BC patients (47), and we found the high expression of *MUC1* was related to high drug sensitivity of tamoxifen, cisplatin, and paclitaxel. Furthermore, the TCGA dataset revealed a negative correlation between the expression of *MUC1* and ICGs and TMB score in patients with breast cancer, indicating that those with low *MUC1* expression may derive greater benefit from immunotherapy. Despite extensive research on MUC1-based immunotherapy over the past few decades, its efficacy remains limited by factors such as the presence of diverse isoforms and immunosuppression (48).

Neuropilin 1 *(NRP1)* is a transmembrane glycoprotein that contributes to cancer development by inducing EMT through various signaling pathways (49, 50). Moreover, it has been reported that *NRP1* is expressed on human pDCs, which contributes to priming immune responses (51), and a positive correlation between *NRP1* expression and pDCs immune infiltrate was found in our study. *NRP1*-mediated immune modulation in cancer has garnered significant attention in recent years (52), and we found that its expression was associated with ICGs, suggesting that it was a promising checkpoint target in BC immunotherapy.

As a member of the matrix metalloproteinase (MMP) family, *MMP11*, also termed stromelysin 3, is overexpressed in BC tissues and BC cell lines, promoting tumor cell proliferation (53, 54). The available evidence suggests that miR-125a directly targets *MMP11*, resulting in downregulation and subsequent suppression of EMT and migration and invasion in osteosarcoma and hepatocellular carcinoma (55, 56). However, the mechanism by which *MMP11* is involved in EMT in BC has yet to be reported. Our study is the first to demonstrate a strong correlation between MMP11 expression and immune cells, ICGs expression, stemness, and drug sensitivity. Furthermore, we have identified MMP11 as a potentially valuable diagnostic marker, with an AUC of 0.993 in the diagnostic ROC curve, with a sensitivity and specificity of 97.3% and 95.2%, respectively. Prior studies have indicated that the combination of *MMP11* and Doppler ultrasound may enhance the diagnostic efficacy for early-stage BC patients (57). These findings propose that *MMP11* could serve as a potential biomarker for precise diagnosis in BC, which deserves our further in-depth study.

Neurotrophic receptor tyrosine kinase 2 *(NTRK2)*, also referred to as *KRKB*, is a constituent of the neurotrophic tyrosine kinase receptors family, serving as a regulator that facilitates EMT by activating PI3K/AKT and IL6/JAK2/STAT3 signaling pathways to foster tumor metastasis (58). Interestingly, while prior evidence has established a correlation between the upregulation of *NTRK2* and its main ligand brain-derived neurotrophic factor (BDNF) with the advancement of cancer progression (59), our current study has yielded contradictory results. Specifically, the expression of NTRK2 was observed to be lower in BC tissues, and patients exhibiting high levels of *NTRK2* expression demonstrated a more favorable prognosis, suggesting that *NTRK2* may play a defensive role in the BC progression. One plausible hypothesis is that *NTRK2* may be involved in the recruitment of immune cells within the tumor microenvironment. Our investigation revealed a positive correlation between *NTRK2* expression and heightened infiltration of CD8^+^ T cells, NK cells, and DCs, all of which are critical components of anti-tumor immunity (60, 61). The precise mechanism underlying *NTRK2*-mediated anti-tumor immunity remains unreported and may differ from the signaling pathway activated by *NTRK2* and BDNF, warranting further investigation.

Laminin γ2 *(LAMC2)* is a subunit of the heterotrimeric glycoprotein laminin-332, which has been demonstrated to facilitate the proliferation and metastasis of cancer cells in basal-like BC (62). It has been observed that the secretion of *LAMC2* by intrahepatic cholangiocarcinoma cells can promote EMT (63). Additionally, *LAMC2* has been shown to regulate gemcitabine sensitivity in pancreatic ductal adenocarcinoma through EMT (64). In the current investigation, we found that *LAMC2* expression in basal-like BC was higher and positively correlated with miR-222-3p expression. Furthermore, we have found a link between higher *LAMC2* expression and reduced drug sensitivity and higher infiltration levels of most immune cells.

Phosphoinositide-3-kinase regulatory subunit 1 *(PIK3R1)* is a regulatory subunit of the PI3K-Akt signaling pathway, which is a tumor suppressor that is frequently downregulated in BC (65, 66). Previous research has shown that under-expression of *PIK3R1* is linked to poor prognosis in BC patients (67). Our study aligns with these findings, as we observed the downregulation of *PIK3R1* in BC and noted that patients with low *PIK3R1* expression had a shorter DSS. Additionally, it has been reported that *PIK3R1* is a target gene of miR-21 and that knockdown of miR-21 leads to upregulation of *PIK3R1*, which in turn inhibits EMT and the activation of the PI3K-Akt signaling pathway, ultimately suppressing BC development (68). Based on the findings of the present and previous studies, we supposed that miR-222-3p might target and downregulate *PIK3R1* expression to promote EMT via the activation of the PI3K-Akt signaling pathway in BC.

Baculoviral inhibitor of apoptosis repeat containing 5 *(BIRC5)* is a mitotic spindle checkpoint gene that belongs to the inhibitor of the apoptosis family, and a mitotic spindle checkpoint gene has been observed to be overexpressed in BC and linked to unfavorable clinical outcomes (69, 70). This study reveals that expression is notably elevated in BC tissues, particularly in basal-like and luminal B subtypes and that *BIRC5* may serve as a valuable biomarker for BC diagnosis, as evidenced by ROC analysis. Patients with high BIRC5 expression levels were found to have a poorer prognosis. Additionally, it has been reported that *BIRC5* can stimulate the expression of superoxide dismutase 1 (*SOD1*) in cancer-associated fibroblasts and transform them into myofibroblasts to promote EMT in BC (71). Furthermore, our investigation revealed a correlation between elevated *BIRC5* expression and reduced levels of NK cells, CD8+ cells, and increased miRNA scores, indicating a potential immunosuppressive function of *BIRC5* and its ability to promote BC cell stemness. Additionally, heightened *BIRC5* expression was linked to the expression of ICGs and TMB, highlighting the potential of *BIRC5* as a promising target for BC immunotherapy.

However, it is important to note that our study primarily relied on data obtained from the TCGA database, which needs further validation. To address the limitations, first, the luciferase reporter assay should be performed to validate the relationship between miR-222-3p and its identified ETGs. Subsequently, it is imperative to conduct further investigation into the EMT-regulated mechanism of the ETGs. Additionally, it is crucial to ascertain whether the ETGs have the potential to serve as therapeutic targets in the future. In conclusion, the findings of this study hold significant implications for future research endeavors aimed at exploring the EMT mechanism of BC, which could potentially yield promising treatment modalities for patients afflicted with BC.

## 5 Conclusion

In conclusion, our study has provided evidence indicating that miR-222-3p was overexpressed in basal-like BC and has the potential to serve as a specific biomarker of basal-like BC. Additionally, elevated expression levels of miR-222-3p in BC are associated with unfavorable prognosis. Through our investigation, we have identified 10 core ETGs of miR-222-3p, among which *MUC1*, *MMP11*, and BIR*BIRC5* may serve as useful diagnostic biomarkers for BC, and *NTRK2*, *PIK3R1*, and *BIRC5* may serve as biomarkers for predicting prognosis. Comprehensive analysis of the association between the expression of 10 ETGs and immune cells, ICGs, TMB, MSI, stemness, and drug sensitivity indicates their potential association with the tumor microenvironment in the progression of breast cancer. These findings suggest that these ETGs may serve as novel therapeutic targets for the treatment of BC.

## Data availability statement

The original contributions presented in the study are included in the article/**Supplementary Material**. Further inquiries can be directed to the corresponding authors.

## Author contributions

YF organized the article writing and critically modified the manuscript. JW and RDZ modified the manuscript. QZ drafted the manuscript and were responsible for the acquisition of data. CC and ZC participated in the data analysis. RJZ contributed to the literature search. CS checked and corrected language expression. All authors read and approved the manuscript and agreed to be accountable for all aspects of the research in ensuring that the accuracy or integrity of any part of the work are appropriately investigated and resolved.

## Glossary

**Table:**

BC	breast cancer
EMT	epithelial–mesenchymal transition
miRNAs	microRNAs
miR-222-3p	microRNA-222-3p
ZEB2	Zinc finger E-box-binding homeobox 2
TCGA	The Cancer Genome Atlas
RPM	read per million
GEO	Gene Expression Omnibus
DEGs	differentially expressed genes
ETGs	EMT-related target genes
GO	Gene Ontology
KEGG	Kyoto Encyclopedia of Genes and Genomes
PPI	protein–protein interaction
FPKM	fragments per kilobase million (FPKM)
TPM	transcripts per million
ROC	receiver operating characteristic
qRT-PCR	quantitative real-time PCR
KM	Kaplan–Meier
OS	overall survival
DSS	disease-specific survival
ssGSEA	single-sample GSEA
ICGs	immune checkpoint genes
TMB	tumor mutational burden
MSI	microsatellite instability
mRNAsi	mRNA expression-based stemness index
OCLR	one-class logistic regression
GDSC	Genomics of Drug Sensitivity in Cancer
HER2	human epidermal growth factor receptor 2
ER	estrogen receptor
PR	progesterone receptor
AUC	area under the curve
NCoR	nuclear receptor corepressor
SMRT	thyroid hormone receptor
HIF-1	hypoxia-inducible factor 1
EGFR	epidermal growth factor receptor
IL6	interleukin-6
STAT3	signal transducer and activated of transcription 3
SERINE1	serine protease inhibitor clade E member 1
NRP1	neuropilin 1
MMP	matrix metalloproteinase
NTRK2	neurotrophic receptor tyrosine kinase 2
BDNF	brain-derived neurotrophic factor
LAMC2	laminin γ2
PIK3R1	phosphoinositide-3-kinase regulatory subunit 1
BIRC5	baculoviral inhibitor of apoptosis repeat containing 5
SOD1	superoxide dismutase 1
